# The Effect of an External Magnetic Field on the Aspect Ratio and Heat Input of Gas-Metal-Arc-Welded AZ31B Alloy Weld Joints Using a Response Surface Methodology

**DOI:** 10.3390/ma13225269

**Published:** 2020-11-21

**Authors:** Pankaj Sharma, Somnath Chattopadhyaya, Nirmal Kumar Singh, Marta Bogdan-Chudy, Grzegorz Krolczyk

**Affiliations:** 1 Department of Mechanical Engineering, Indian Institute of Technology (ISM), Dhanbad 826004, India; shampan3@rediffmail.com (P.S.); somuismu@gmail.com (S.C.); nks_221@yahoo.co.in (N.K.S.); 2 Department of Manufacturing Engineering and Automation, Opole University of Technology, 45758 Opole, Poland; m.bogdan-chudy@po.edu.pl

**Keywords:** aspect ratio, AZ31B, EMF, GMAW, HI_f_, RSM

## Abstract

This study attempted to analyze and optimize the effect of an external magnetic field (EMF) on the aspect ratio and heat input for AZ31B weld joints that were welded using the gas metal arc welding (GMAW) process. The response surface methodology (RSM) was adopted for the critical analysis, and subsequently, mathematical models were developed based on the experimental results. It was observed that the EMF and its interaction with the wire feed rate significantly affected the aspect ratio and heat input, respectively. At 119 G (magnetic field), 700 mm/min (welding speed), 5.8 m/min feed rate, and 11.5 L/min (gas flow rate), the aspect ratio was 2.26, and the corresponding heat input factor (*HI_f_*) was 0.8 with almost full weld penetration.

## 1. Introduction

Nowadays, petrochemical fuel availability, global warming, and environmental pollution are critical issues for debate and discussion. The Environmental Defense Fund (EDF) estimates that on-road vehicles cause one-third of air pollution and are responsible for 27% of greenhouse gas emissions. This serious environmental issue can be controlled via intentional and valid cuts in vehicle weight. An approximately 100 kg cut in vehicle weight can reduce fuel consumption by 5% (magnesium alloy manual). Furthermore, a 10% reduction in average vehicle weight can lead to reductions in CO_2_ emission by approximately 13% [1].

Magnesium alloys have some inherent characteristics, such as low weight and compatible specific strength compared to its market competitor, namely, cold-rolled carbon–hot rolled carbon (CRC–HRC).

The availability and recyclability make it a “green material” of the 21st century. Magnesium is ranked as the eighth-most abundant element on the Earth’s surface, and fifth in seawater [2]. However, its commercial viability in vehicle manufacturing depends upon good quality weld joints [3]. It is predicted to be one of the most used materials for next-generation automobiles to substitute CRC–HRC and other non-ferrous alloy sheets. Magnesium alloys (AZ31B) would enable us to achieve weight reduction and fuel efficiency. To attain these features, having reliable and robust methods of assembly of adequate numbers of such magnesium (AZ31B) components in an economically viable way is required. Magnesium alloy (AZ31B) is an exotic metal for coalescence via the fusion welding process and calls for appropriate control of the heat input and arc stability. Out of various metal joining processes, metal inert gas (MIG) or gas metal arc welding (GMAW) has been recommended as an economical and suitable process for joining metals used in the body-in-white (BIW) parts due to them having high melting points, and soon, no other process can replace this process on the grounds of economic production and ease of use. However, GMAW in its present state is unsuitable for welding magnesium alloys.

Thus, to avoid the challenges offered by the GMA process for the joining of magnesium alloys and to deny any possibility of the replacement of this joining process for welding magnesium (AZ31B), a process modification is desired. Chai et al. [4] observed that the problems of welding come up as a distorted bead of magnesium alloy while performing GMA welding on butt joints due to uncontrolled heat input. Zhang et al. [5] observed large size spatter at high heat input with globular and spray transfer for joining magnesium alloys. The low melting and boiling point of the AZ31B alloy may be the reasons for the distorted bead or large size spatter while joining using the GMA welding process. Thus, the GMA welding process needs better control of the heat input and arc stability while welding magnesium alloys.

The past research in the direction of controlling the arc density and stability using an additional magnetic field (EMF) opens the scope for this process and material in a way to get good welds. The susceptibility of the arc toward the external magnetic field has led to extensive investigations over the past many years. The debate over the dynamic effect of the magnetic field was started with Sir Michael Faraday (1821) and states that magnetic fields generate a directional force on the flow of electrons (electric current); with time, the concept switched from the macrolevel (electric motors and generators) to the microlevel in a way to control the flow of electrons or arc plasma to regulate the arc shape and arc density.

Its application in the field of welding around the weld zone was initiated in circa 1962. The study estimated the effect of a magnetic field on the molten metal pool. Since 2000, a magnetically controlled welding method has widely been used in inert gas welding {tungsten inert gas (TIG)/metal inert gas(MIG)/metal active gas(MAG)}, resistance welding, submerged arc welding, laser beam welding, and hybrid welding. Therefore, this type of controlled arc welding is a favorable welding process. Bachmann et al. [6] found that the EMF controls the flow of the metal, the weld pool temperature distribution, and the shape of the weld cross-section. The supplementary electromagnetic force alters the flow conditions of the molten metal. The optimized magnetic field intensity can effectively diminish the charged particle density by altering moving charged particles under the Lorentz force. Nomura et al. [7] investigated permanent magnets’ effect on the plasma arc shape of TIG welding arc and observed a controlled weld arc and subsequently achieved a sound weld shape.

However, an uncontrolled external magnetic field may lead to a poor weld. Thus, an optimized EMF has been strongly recommended for process modification due to the widely accepted susceptibility of the arc to an EMF. The design of experiment (DOE) with the central composite design has been investigated in detail by many researchers, such as Montgomery [8] and Giridharan [9], for the optimization and the development of the mathematical model.

In previous experiments [10], as a strategic process modification, an EMF has been used to control the mechanical properties and microstructure of GMA butt welds. This experimental work intended to analyze the effect of an EMF on the bead geometry in terms of the aspect ratio (*AR*), which is based on the bead width (*W*) and bead penetration (*P*), along with the development of robust mathematical models to estimate the optimized conditions. This dimensionless number can be directly associated with the weld quality. A higher value signifies a poor weld penetration depth (*P*) or a large bead width (*W*).

Simultaneously, there is no known mathematical relation between the heat input and the external magnetic field. Thus, based on statistical data, mathematical modeling was done to relate the heat input with the external magnetic field. In this study, the heat input factor (*HI_f_*) was used as another response parameter instead of the heat input to interpret the effect on the bead geometry in terms of the minimum aspect ratio (*AR*). The experimental results obtained were used for statistical modeling and a study of the influence of the welding process parameters on the bead geometry characteristics, including the aspect ratio (*AR*), bead penetration (*P*), and bead width (*W*), along with the heat input.

## 2. Materials and Methods

### 2.1. Materials of the Plates and Wire 

**Figure 1:**
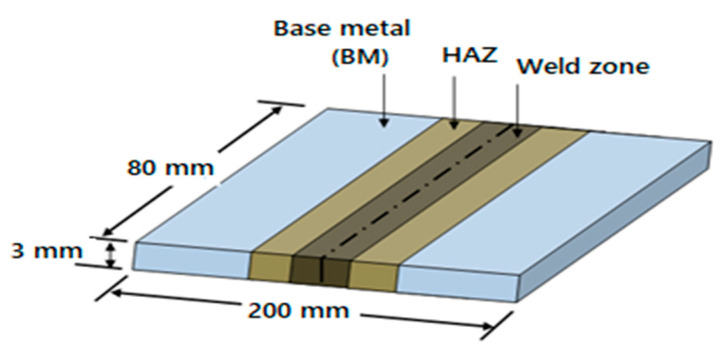
Weld specimen, HAZ (heat-affected zone).

The specimens were dissected using wire cut EDM(electric discharge machine), per the ASTM E140-05e1 standard [11]. The specimen surfaces were cleaned with acetone, sandpaper, and a wire brush to remove impurities. The filler wire used was ER-AZ61A, with a diameter of size 1.6 mm and an aluminum content of 5.8–7.2%, compared to the 2.5–3.5% of AZ31B to avoid intermetallic weld cracking [2,10]. The magnesium alloy (AZ31B) thin plates were welded as a square butt joint, as shown in Figure 1.

### 2.2. Experimental Setup

**Figure 2:**
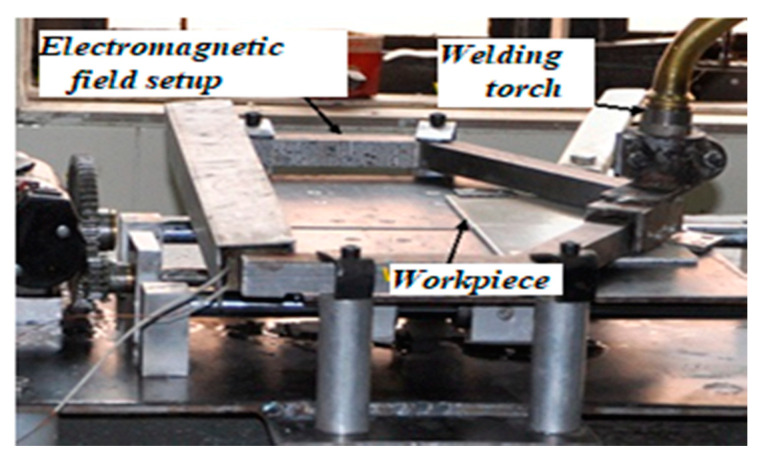
Schematic of the experimental setup [10].

The experiments were performed on a constant voltage digital power source EWM-Taurus-505 GMAW welding machine (Mundersbach, Germany) in the presence of EMF by applying an electromagnetic setup that was designed and fabricated to generate a magnetic zone around the weld. The magnetic field’s intensity was measured using a digital gauss meter (DGM-102, range 0–2.0 T, Roorkee, Uttarakhand, India). The whole setup for the experimentation is shown in Figure 2.

### 2.3. External Magnetic Field and the Arc Shape

Yibo et al. [12] discussed the effect of an EMF on a welding arc in metal arc joining in terms of the longitudinal external magnetic field (LEMF) or a transverse external magnetic field (TEMF). The EMF inevitably changes the intensity of the magnetic field around the arc, resulting in a change in the arc’s shape. The magnitude of the EMF and the change in the arc shape are causally related to each other. The EMF strength depends upon the excitation current. Wang et al. [13] discussed the effect of an EMF on the backward flow of molten metal during welding and controlled the humping bead issue by increasing the welding speed and cooling rate.

#### 2.3.1. Longitudinal and Transverse External Magnetic Fields

Naomi et al. [14] discussed the effect of alternating the LEMF and TEMF on the arc behavior. In the LEMF, the EMF is in the direction of the weld, as well as at right angles to the axis of the welding arc, while in the TEMF, the EMF is at right angles to both the axis of the welding arc and the weld direction.

Shuyuan et al. [15] introduced an adscititious longitudinal magnetic field to control the CO_2_ welding process, believing that the welding arc can act with the magnetic field. The results show that the optimal excitation current resulted in a smaller droplet size and a stable welding process without an adscititious magnetic field. The tangential electromagnetic force (Ft) due to the TEMF on the metal drops from electrode wire resulted in an elongated ellipsoid shape.

However, many characteristics of the TEMF are like the LEMF. In this investigation, a TEMF was used so that the Lorentz force acted in the direction of weld to elongate the arc shape along weld length such that the heat-dissipating area increased along the weld length but constricted the width. The optimal EMF was favorable for keeping the aspect ratio (*AR*) at a minimum. A suitable magnetic field also suppressed the weld flow, reduced the spatter, and produced an acceptable weld appearance. The EMF increased the magnetic density, supported the disconnection of the short circuit liquid bridge, and decreased the instantaneous short-circuiting spatter. The intensity and direction of the magnetic field had a significant influence on the welding quality and heat input only when the parameters of the EMF, namely, the welding speed (*S*), wire feed rate (*F*), and gas flow rate (*G*), were matched optimally. The strength of the magnetic field is usually expressed in terms of the magnetic flux density measured in units of gauss (G) or milli-gauss (mG) (1 gauss = 10^−4^ tesla).

#### 2.3.2. Heat Input Factor and the Transverse External Magnetic Field

Given the low melting point and boiling point of AZ31B, control of the heat input plays a vital role when controlling the weld quality. There is no direct mathematical relation to relate the external magnetic field (*M*) with heat input. Thus, control over the heat input due to an external magnetic field (*M*) along with the relative influence of the wire feed rate (*F*), welding speed (*S*), and gas flow rate (*G*) was generalized. The heat input is an incredibly vital aspect, which affects the bead geometry and characteristics of the weld. The effect of the welding speed (*S*) and feed rate (*F*) on the heat input per unit length is given below:

(1) $$H\;\propto \;I\;\propto \;\frac{1}{S},$$

(2) F ∝ I.

To improve the control over this heat input in the weld zone, Nosov and Peremitko [16] studied the effect of an EMF on the heat of the weld zone and established the heat source model and the molten pool model. This EMF exerts a force on the arc in a forward direction, making the arc an elliptical shape. As a result, the heat-dissipating area increases, and heat input per unit length decreases. It was found that under an EMF, charged particles of arc plasma experience a force known as the Lorentz force, which is given by one of Maxwell’s equations (Equation (3)) as:

(3) F=q(V×M),

where *F* is the Lorentz force, *q* is the charge on particle, *V* is the velocity of the particle, and *M* is the EMF.

**Figure 3:**
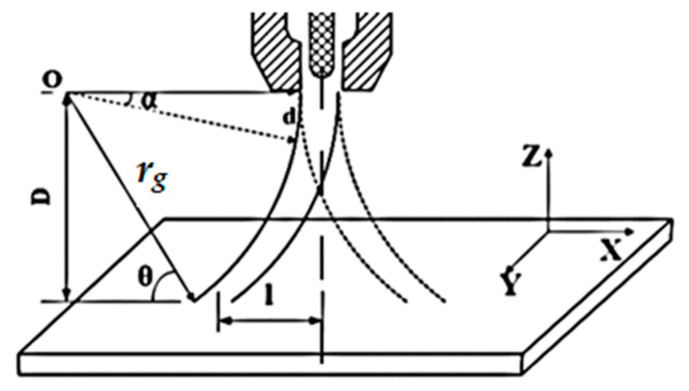
The magnetic field is about the Y-axis, the Lorentz force about the X-axis, r_g_ is the radius of gyration (i.e., movement of the arc plasma) [17].

Figure 3 shows the path of charged particles of an arc plasma under an EMF shown about the Y-axis. The resultant Lorentz force will be about the X-axis, as the direction of charged particles of arc plasma is perpendicular to both the velocity and magnetic field (as *V* and *M* are in a cross product), as described in the Equation (3).

This results in the gyration of the particles (i.e., they move in a circle) and may change the arc shape and arc density, and thus, the temperature gradient. This gyro-radius or radius of the circle (*r_g_*) can be obtained by comparing the Lorentz force of Equation (3) with the centripetal force on a charged particle, as shown in Equation (4):

(4) $$q\left(V\times M\right)=\frac{M_{p}V^{2}}{r_{g}},$$

where *M_p_* is the mass of the charged particle and *r_g_* is the radius of gyration. Therefore, for any charged particle, if the magnetic field (*M*) increases, the radius of gyration (*r_g_*) decreases, as shown in Equations (5) and (6):

(5) $$r_{g}=\frac{M_{p}V}{qM},$$

(6) $$r_{g}\propto \frac{1}{M}.$$

Furthermore, the radius of gyration (*r_g_*) directly affects the length (*l*) of the arc on the workpiece, as shown in Equation (10). The relation is obtained using a simple geometrical calculation with reference to Figure 3 and using Equations (7)–(9). To get the relation between the arc radius (*r_g_*), the arc length on the workpiece (*l*), and the distance between the top of the workpiece and the bottom of the torch (*D*), from the geometry shown in the figure, for any angle *θ* using Pythagoras’s theorem:

(7) $$r_{g}^{2}=D^{2}+X^{2},$$

or:

(8) $$X^{2}=r_{g}^{2}-D^{2},$$

(9) $$X=\sqrt{r_{g}^{2}-D^{2}},$$

where $X\;=r_{g}^{2}\;-\;l$, thus $l=r_{g}^{2}-X$:

(10) $$l=r_{g}^{2}-\sqrt{r_{g}^{2}-D^{2}}.$$

**Figure 4:**
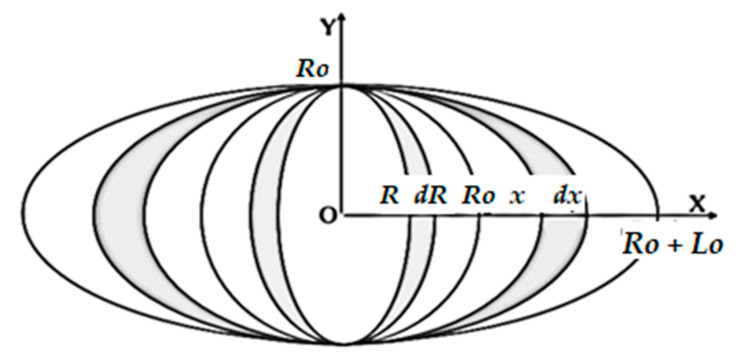
Length of a plasma arc in an external magnetic field [17].

Therefore, from this relation between the arc length (*l*), arc radius (*r_g_*), and *D*, it is observed that for a given value of *D* when the radius of gyration (*r_g_*) reduces from the radius of an infinite circle for an arc without an external magnetic field to a radius of gyration (*r_g_*) equal to *D*, then *l* will be at a maximum. The arc radius (*r_g_*) cannot be reduced to less than *D* or the arc will be unstable. The length of the arc on the workpiece is inversely proportional to the magnetic field. Figure 4 depicts the length of the arc (*l*) on the workpiece under the influence of an EMF and without an EMF.

Ghosh et al. [18] observed the elliptical shape of the heat distribution on the welded plate for the SAW (submerged arc welding) process for a particular input parametric setting that was measured in terms of the dimensions of the bead geometry. Furthermore, Ghosh et al. [19] observed that the temperature distribution is essential for controlling the heat-affected zone (HAZ) dimensions and to get the required bead size and quality in the SAW process, which was achieved by using an analytical solution for moving the heat source with a Gaussian distribution of the inside volume of the central conical shape.

Ghosh et al. [20] also attempted to find out the analytical solution of the thermal field induced in a semi-infinite body by a moving heat source with a Gaussian distribution by selecting an appropriate inside volume for the SAW process. The study showed that for the submerged arc welding process for the heat input, the best suitable heat source shape is in the form of an oval. Kumar et al. [21] investigated the temperature-dependent properties on a commercial 3D finite element model and observed an appreciable agreement between the experimental and the simulated temperature fields in most cases.

Meng and Dong [17] developed a mathematical model for heat input (*Q_in_*), shown in Equation (11), which states that the heat input is inversely proportional to the arc length on the workpiece:

(11) $$Q_{in}=\iint \frac{T.W}{2l_{o\;}R_{o}}dR.dx,$$

where *T* is the time, *W* is the total heat flux, *R_o_* is the radius of the arc without an EMF, *l* is the length of the arc on a workpiece from the center of the arc (*R_o_* + *l_o_*).

Because the increase in *l_o_* increases the heat distribution area, under boundary conditions keeping arc stable, the heat input per unit length is inversely proportional to the EMF. Thus, under boundary conditions, the EMF increases the heat-dissipating area, thus reducing the heat input per unit length, as shown in Equation (12):

(12) $$H\propto \frac{\;\;1}{M}$$

Furthermore, the flow rate of the inert gas affects the density of the inert environment over the weld. The shielding by the ionized inert gas hinders the heat dissipation rate. As an increased gas flow rate accelerates the plasma velocity, the radius of the heat flux distribution decreases. Thus, the heat flux of the plasma arc on the workpiece surface is more concentrated with an increase in the gas flow rate. Therefore, the shielding of the inert gas over the weld area increases the heat input per unit length, as shown in Equation (13):

(13) H ∝G.

The combined influence of these variables on the quantity of the heat input is taken as the response heat input factor (*HI_f_*), where there is a lack of an analytical equation for this combination. Based on experimental data, a general equation was formulated, as shown in Equation (14):

(14) $$HI_{f}\propto F\;\propto \frac{1}{S}\;\propto \frac{1}{M}\;\propto G$$

or:

(15) $$HI_{f}=K\;\frac{F\times G}{S\times M}.$$

A higher aspect ratio (*AR*), large size bead width (*W*), burn out, evaporation of alloying elements, porosity, and weld crack are the side effects of unoptimized heat input. Thus, just like the aspect ratio (*AR*) number, this heat input factor (*HI_f_*) number may also reflect some information about the weld quality and optimization. Thus, the heat input factor (*HI_f_*) as a dimensionless number shows the cumulative effect of the process parameters (*F*, *M*, *S*, and *G*) on the aspect ratio (*AR*) for the bead width (*W*) and penetration depth (*P*). Thus, data were recorded for the heat input factor (*HI_f_*), per Equation (15).

Further statistical modeling and optimization were done for Equation (15) to relate the outcomes of this response with other weld characteristics. The results so obtained were analyzed based on the criterion that heat input was increasing or decreasing for a given aspect ratio (*AR*). From a literature study and pre-investigation experiments, the boundary conditions obtained for these process parameters were divided into five levels for the further design of a matrix to perform experiments, which are shown in Table 1 with levels and ranges.

### 2.4. Response Surface Methodology (RSM)

The RSM has been used for experimentation and optimization of this multi-objective process due to the widely known application and characteristics of the DOE, with four factors and five levels. This helps to reduce the number of experiments, achieve an optimal process output, and minimize, maximize, or target the output in any statistical and experimental study to predict responses. The DOE was used to produce 31 sets, including 16 factorial designs, 7 center runs, and 8 star runs to optimize the experimental conditions. The second-order statistical model for the process parameters was developed in the following form of a general equation [8], as shown below:

(16) $$Y_{ij}\;=\beta_{0}+\;\overset{k}{\underset{i=1}{\sum }}\beta_{i}X_{i}+\;\overset{k}{\underset{i=1}{\sum }}\beta_{ii}X_{i}^{2}+\;\sum \underset{i<j}{\sum }\beta_{ij}X_{i}X_{j}+\;\varepsilon_{ij}$$

where *Y_ij_* is the expected process yield, $\beta_{0}$ *is constant regression coefficient, β_i_* are regression coefficients, *X_i_* and *X_j_* denote the direct process parameters, *k* stands for the number of variables, and *ε* is the inherent experimental error.

The run order design matrix was used to perform the experiments. In this welding experiment, popular butt weld joints were considered to be welded as the plate thickness was less than 5.00 mm, where a square groove butt joint was used for experimental work. The literature recommends a 0.80 mm to 1.0 mm gap for a square groove. Since a single-pass, full penetration weld is recommended for thin plates, the same was applied here. A groove of depth 1.5 mm on the base plate as a backing plate helped to control the bead formation on the rear side, which is recommended in the ASME *(American Society of Mechanical Engineers)* handbook. The specimen surfaces were cleaned with acetone, sandpaper, and a wire brush to remove any traces of oil, oxides, and other impurities before welding. Furthermore, rough edges due to the shearing action on workpieces were smoothened using sandpaper for a better result.

Since magnesium alloy (AZ31B) has a low melting point (660 °C) and boiling point (1100 °C), care was taken to avoid burning and vaporizing the molten metal. For this reason, a low arc voltage is preferred, i.e., 18 V. Dong et al. [22] performed experimentation with a 26 V arc voltage without a magnetic field and reported porosity and weld cracks. Thus, in this experiment, the arc voltage was kept at around 18 V, which considerably reduced the heat input by about 25% and induced localized heating of the workpiece under EMF. Therefore, a weld with deep penetration, even at a low heat input, was observed. This trend was found to be consistent with the earlier findings by Nomura et al. [7], while controlling the shape of the arc in TIG welding.

**Figure 5:**
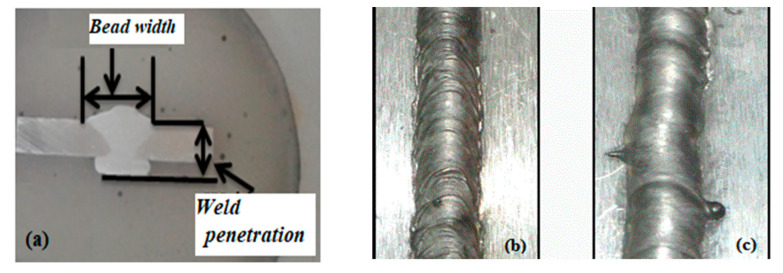
(**a**) Macrostructure of the bead and (**b**) the bead profile without a magnetic field and (**c**) with a magnetic field.

Furthermore, 31 weld specimens were machined on the wire cut machine to prepare for the measurement of the corresponding responses due to the geometrical characteristics, as shown in Figure 5a.

Figure 5b,c shows a typical weld bead contour, showing the magnetic field’s influence on the bead’s surface. The bead’s surface provides a vital sign of the process’s steadiness and can be seen by bare eyes. A smooth surface denotes a steady process, while an uneven and irregular surface indicates a flawed process.

The further dimensions of the weld bead geometry, including the bead width (*W*) and bead penetration depth (*P*), were measured and recorded with the help of built-in linear measuring devices in the optical microscope with an accuracy of 0.001 mm. Later, aspect ratios (*AR*s) based on the bead width (*W*) and bead penetration (*P*) were calculated and recorded, as shown in Table 2.

### 2.5. Development of the Mathematical Model

The mathematical models for the responses were developed and modified using the backward elimination method to remove insignificant terms from the model’s equations. The terms with their probability level (*p* > 0.05) (more than 5%) were removed from the model. The regression analysis was again carried out and the adequacy of the model was checked. Equations (17)–(20) show the new second-order polynomial regression models for the responses.

(17) $$AR\;=\;126.6\;-0.1181M-26.33F-0.1146S+0.158G+0.001143M^{2}+1.769F^{2}\;+0.1109G^{2}-0.02300M\times F+0.01735F\times S-0.3575\;F\times G,$$

(18) $$HI_{f}=0.01183M+0.595F+0.00357S-0.172G-0.00317M\times F-0.000825F\times S\;+0.0412F\times G-1.79,$$

(19) $$P=-54.66\;+0.0528M\;+11.99F+0.0595S-0.2808G-0.000605M^{2}\;-0.605F^{2}\;+0.01383M\times F-0.00925\;F\times S,\;\;$$

(20) W=−31.9 –0.2047M +4.01F +0.0976S+1.743G+0.03267M×F−0.0112F×S−0.00315 S×G. 

#### 2.5.1. Acceptability of the Developed Models

The outcomes of the ANOVA regression models for heat input factor (*HI_f_*) and aspect ratio (*AR*) in terms of the F ratio (74.91 and 125.36) compared with the tabulated F ratio values (F_0.05,10,20_ = 2.34 and F_0.05,7,23_ = 2.44) showed that the observed values of the F ratio were found to be more than the tabulated values. While for a lack of fit for the *HI_f_* and *AR*, the observed values of the F ratios (1.83 and 1.87) were less than the tabulated values (F_0.05, 17,23_ = 2.28 and F_0.05, 14,20_ = 2.22); thus, the lack of fit was insignificant. Furthermore, multiple correlation coefficients R^2^, adjusted for *HI_f_* and *AR*, were 94.52 and 97.64%, respectively. Thus, the models were adequate. Equation (21) estimates the precision of the errors to predict the accuracy of the models, including errors that occurred due to random reasons. The uncertainty for the responses were ∆*AR* = ±0.35, ∆*HI_f_* = ±0.08, ∆*P* = ±0.37, and ∆*W* = ±0.42, respectively.

(21) $$\Delta t=\pm t_{\alpha /2,\mathrm{DF}}\sqrt{V_{e}}$$

where ∆*t* is the error range of predicted models, *t* is the statistical value of horizontal coordinate on the *t* distribution corresponding to the specified degrees of freedom, *α*/2 is the level of the confidence interval, DF represents the degrees of freedom, i.e., the value of the horizontal coordinate of the *t* distribution, *α* is the level of the confidence interval, and *Ve* is the variance of the residual error of the predicted model.

#### 2.5.2. Validation of the Model

Furthermore, experiments were performed to confirm the accuracy of the models. For the validity tests, the process parameters were selected randomly in the given range, and tests were performed. The values of the process parameters and responses are listed in Table 3. It was seen that the models could predict the responses and the experimental results were within the error range calculated using Equation (21).

#### 2.5.3. Optimization of Bead Parameters

The responses of the welding process were multi-objective and the responses, such as the *HI_f_*, *AR*, *P*, and *W*, were related to a high-quality bead; thus, the optimal solution found a middle ground. The aspect ratio (*AR*) depended upon the bead width (*W*) and the weld penetration (*P*) ratio. In general, for the optimal value of *AR*, the boundary condition varied between the minimum bead width (*W_min_*) and the maximum weld penetration depth (*P_max_*), and in this experiment, the bead width (*W*) should be more than the diameter of the filler wire (1.6 mm) and the penetration depth should be greater than or equal to the plate thickness (3 mm). Thus, lower values of *AR* signified a good penetration depth (*P*) that led to better bead geometry.

Theoretically, the process parameters were optimized using the software tool “optimizer,” which is available in Minitab (17, State college, PA, USA), with the objective function as the aspect ratio (*AR*) to be minimized, as shown in Equation (22), keeping an eye on the heat factor, as shown in Equation (23). The corresponding inputs are shown in Equation (24).

(22) $$\begin{array}{ll} Minimizef\left(x\right)= & AR\\ & =\;126.6-0.1181M-26.33F-0.1146S\;+0.158G\\ & +0.001143M^{2}\;+1.769F^{2}+0.1109G^{2}-0.02300M\times F\\ & +0.01735F\times S-0.3575\;F\times G \end{array}$$

(23) $$\begin{array}{ll} MInimizef\left(x\right)= & HI_{f}\\ & =0.01183M+0.595F+0.00357S-0.172G-0.00317M\times F\\ & -0.000825F\times S+0.0412F\times G-1.79 \end{array}$$

(24) given  90 ≤M≥150,  5.0 ≤F≥7.0,  500≤S≥700,  9≤G≥13

The predicted and optimized results of the process parameters using the response “optimizer” tool were *M* as 119 G, *F* as 5.8 m/min, *S* as 700 mm/min, and *G* as 11.5 L/min. The predicted responses were 0.8 for the *HI_f_*, and 2.4 for the *AR*. Furthermore, the GMA welding parameters were set close to the optimized combination and experiments were performed. The experimental result for the aspect ratio was 2.26 with a corresponding full penetration.

## 3. Results and Discussion

The developed model correlated the input variables with the bead characteristics and heat input factor for magnesium alloy welds. The direct and interaction outcomes of the parameters on the bead geometry were analyzed.

### 3.1. Effect of the EMF (M) on HI_f_, AR, P, and W

**Figure 6:**
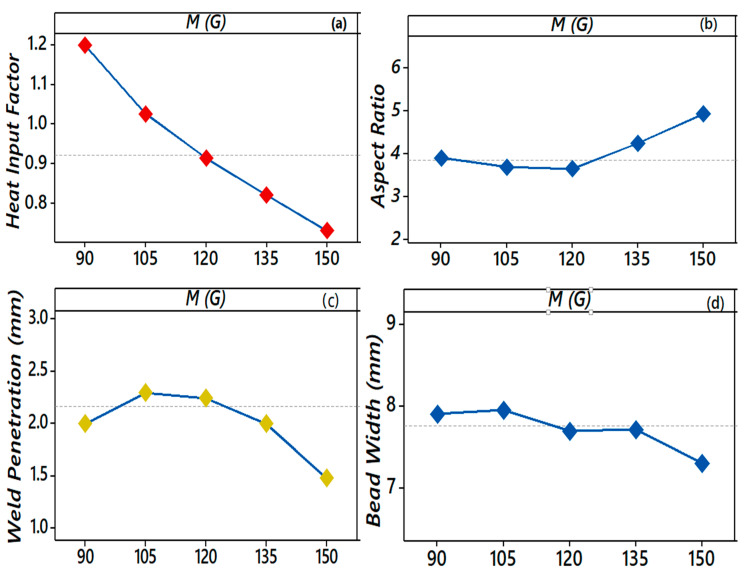
(**a**–**d**). Effect of the EMF on the *HI_f_*, *AR*, *P*, and *W* while keeping *F*, *S*, and *G* at 6 m/min, 600 mm/min, and 11 L/min.

The functional behavior of the EMF on *HI_f_*, *AR*, *P*, and *W* is shown in Figure 6a–d. The *HI_f_* decreased as the magnetic field (*M*) increased. It decreased steadily for all values of the magnetic field (*M*) from 1.2 to 0.7. The higher values of *M* negatively affected the bead characteristics, as initially, the penetration depth (*P*) was low, increases till 120 G for *M*, and then again, *P* started reducing. The variation in *AR* (3.9 to 3.5) was not significant until *M* reached 120 G, because in this range, *P* increased while the change in the bead width (*W*) was insignificant or decreased.

A further increase in *M* beyond 120 G caused the decrease in *W* to be more significant than *P*, and hence *AR* varied substantially. *P* increased from 2 to 3 mm till the *M* reached 120 G, but beyond 120 G, it decreased. The effect of *M* on *W* was nearly the same from 90 to 105 G, and a further rise resulted in a reduced *W* till 150 G.

The EMF controlled the density of the charged particles in the arc column by reducing vaporization. It gave a higher deposition rate, controlled the deviation in the arc column, and restricted the variation in *W*. However, at the elevated EMF, the arc column was restricted more, resulting in a higher arc instability near 150 G. Figure 5 compares the weld appearance with and without the EMF. From Figure 5b, it is observed that the weld appearance was rough and erratic layers were observed at a very short distance. Figure 5c shows a good wetting behavior, which resulted in a smooth weld appearance when the EMF was applied. A low heat input effect was found from the weld cross-section, demonstrating an inferior average heat input into the AZ31B sheet throughout the welding process.

**Figure 7:**
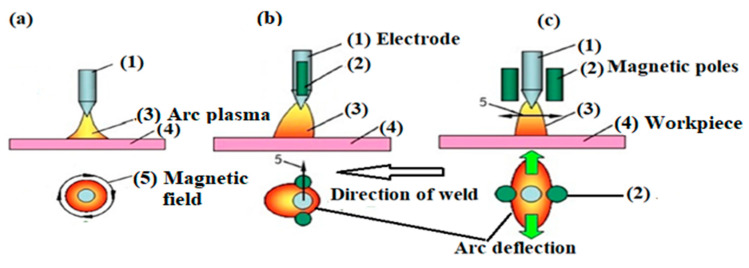
Weld arcs (**a**) without a magnetic field (**b**) with the TEMF, and (**c**) with the LEMF [23].

The Lorentz force of the EMF (LEMF or TEMF) generates turbulence in the molten pool, as shown in Figure 7b,c compared to Figure 7a with no external field, which accelerates the heat dissipation rate along the weld length in the molten pool due to constriction of the arc in the vertical direction, resulting in the elliptical shape of the arc along the weld length. As the external magnetic field intensifies, the resultant force also increases and the deflecting magnitude of the welding arc increases.

**Figure 8:**
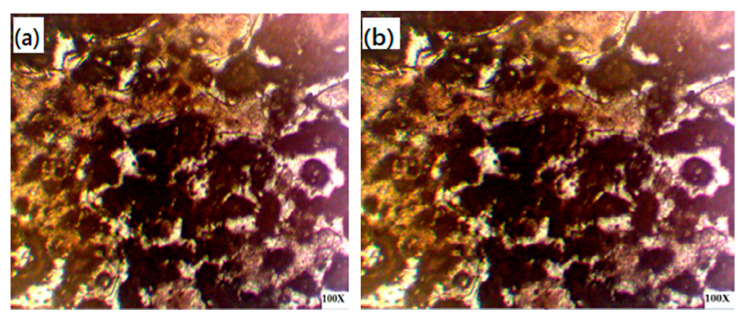
Optical micrograph of the weld zone: (**a**) with the magnetic field and (**b**) of the base metal [10].

This localized heating of the workpiece resulted in deep penetration and a low bead width in a weld joint even at a low heat input. Furthermore, the temperature gradient influenced the cooling rate and grain size, and under a forced weld arc deflection, significant changes were observed in the microstructure, as shown in Figure 8a,b. The EMF influenced the weld metal’s surface, thus increasing the effective contact area and the stability of the welding process was improved. As the magnetic field strength incrementally increases, the resultant force is increased, and the deflecting magnitude of the welding arc also increases [24]. Reddy et al. [25] adroitly demonstrated the effect of an external magnetic field on grain refinement in gas tungsten arc (GTA) welding.

### 3.2. Interaction Effect of the EMF (M) and F on the HI_f_, AR, P, and W

The three-dimensional (3D) and two dimensional (2D) plots show the dimensional relationship between the responses and variables. These plots are also known as interaction effect plots. Here, the 2D plot shows the behavior of a 3D plot through the cut section to get a deep insight into the relationship between the magnetic field (*M*) and wire feed rate (*F*) for the corresponding responses of the heat input factor (*HI_f_*), aspect ratio (*AR*), weld penetration (*P*), and bead width (*W*).

**Figure 9:**
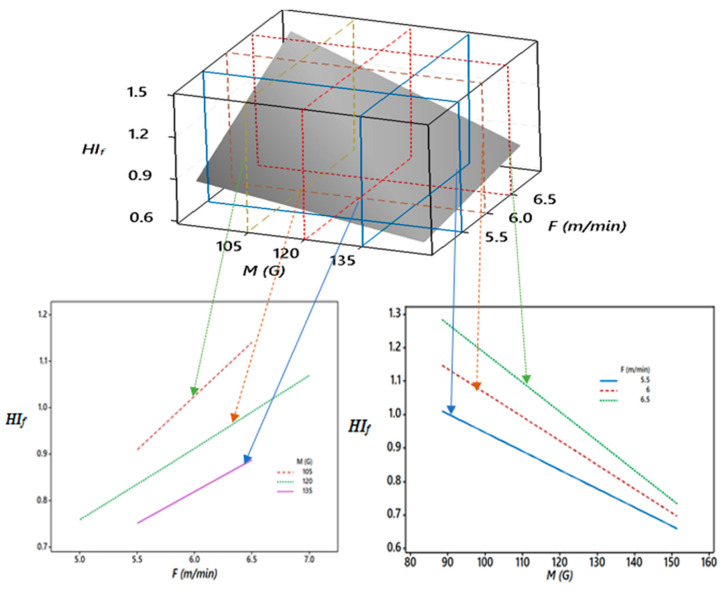
Sectioned 3D and 2D plots for the interaction effects of *M* and *F* on *HI_f_*.

Figure 9 shows the critical 3D and 2D relationships between the heat input regulation (*HI_f_*), magnetic field (*M*), and wire feed rate (*F*). It was observed that the heat input factor (*HI_f_*) continuously decreased as the magnetic field (*M*) increased from 90 to 150 G and higher values of feed rate (*F*) resulted in a higher *HI_f_* for given values of *M*. Thus, for the different combination of feed rates (*F*) and magnetic fields (*M*), *HI_f_* varied from 0.7 to 1.3.

**Figure 10:**
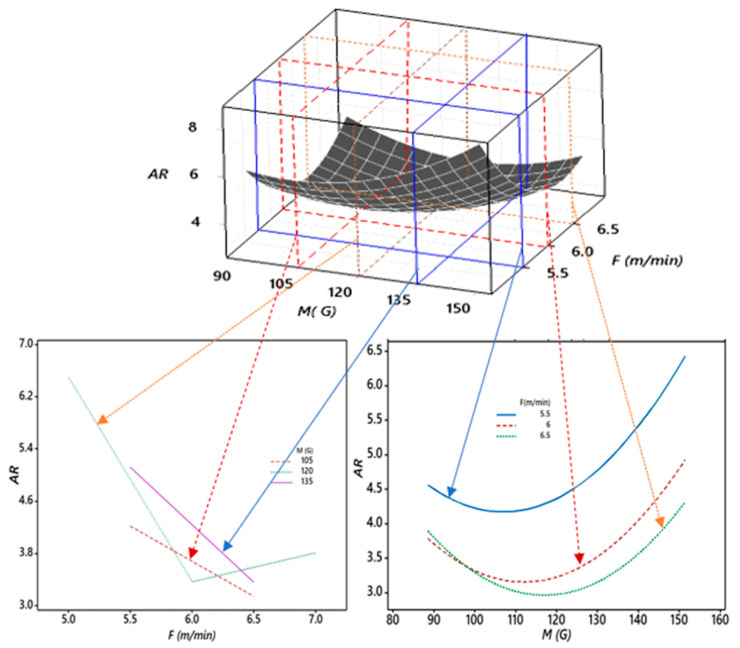
Sectioned 3D and 2D plots for the interaction effects of *M* and *F* on *AR*.

Furthermore, Figure 10 shows the critical 3D and 2D relationships between the aspect ratio (*AR*), magnetic field (*M*), and wire feed rate (*F*). It was observed that as *M* increased from 90 to 110 G, *AR* decreased for all values of *F*. The slope of *AR* was negative until *M* was 110 G. However, beyond 110 G for *M*, *AR* started increasing, and the slope was positive and steeper. For a good quality weld, a low value of *AR* is considered a desirable quality. Thus, this undesirable rise in *AR* may hamper the quality of the weld, and it was because of the effect of the higher magnetic field (*M*) on the arc density and the effect of the wire feed rate (*F*) on *HI_f_*. It also resulted in significant changes in the bead geometry. At an *F* of 5.5 m/min, *AR* was about 4.5 at an *M* of 90 G, but as *F* increased, *AR* decreased to 3.0 at an *M* of 120 G. Thus, experimentally, it was observed that higher *F* values till 120 G for *M* were desirable for the good quality weld. However, beyond 120 G, the objective function used to minimize the *AR* was hampered.

**Figure 11:**
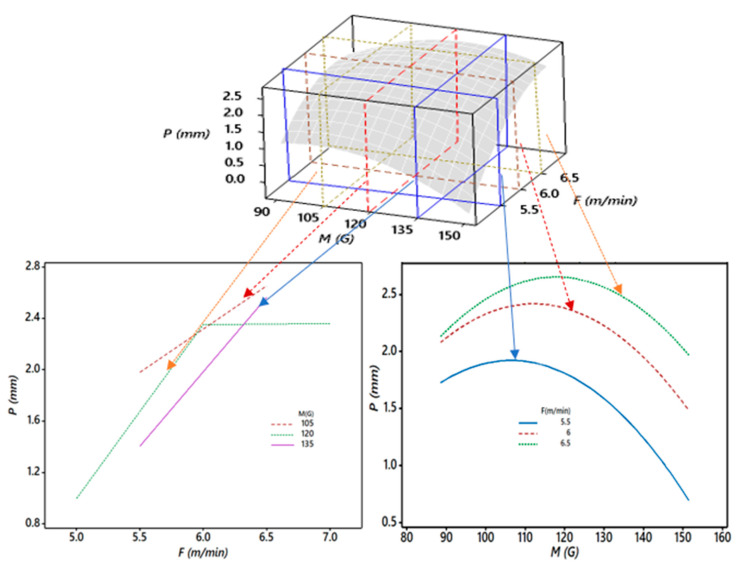
Sectioned 3D and 2D plots for the interaction effects of *M* and *F* on *P*.

Furthermore, Figure 11 shows the critical 3D and 2D relationships between the weld penetration (*P*), magnetic field (*M*), and wire feed rate (*F*). It was observed in Figure 10 that as the magnetic field (*M*) increased from 90 to 120 G, the weld penetration (*P*) increased significantly for all values of the wire feed rate (*F*). The further rise in *M* resulted in a decreasing trend of the *P* curve. It was at a maximum when *F* was a maximum and *M* was in the range of 110–120 G, where the minimum occurred when *M* was at a maximum and *F* was at a minimum. The changes in *F* relative to *M* from 5.5 to 6.5 m/min significantly changed *P*.

**Figure 12:**
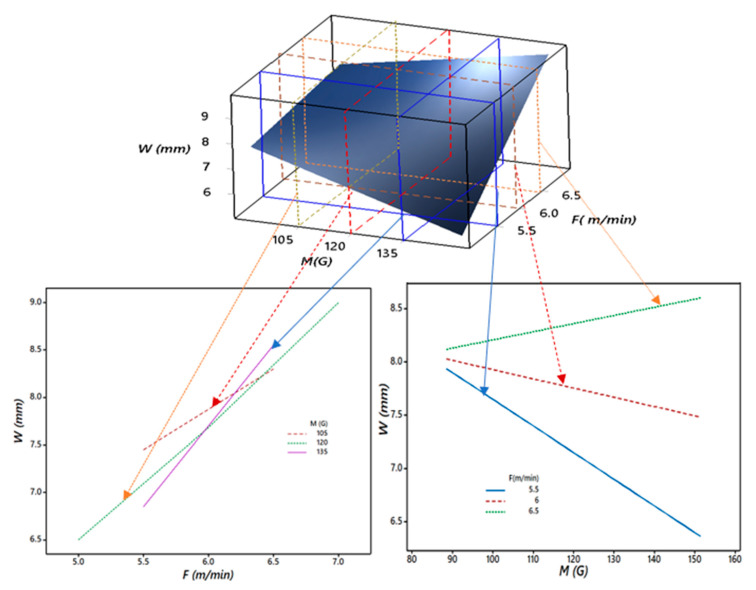
Sectioned 3D and 2D plots for the interaction effects of *M* and *F* on *W*.

Furthermore, Figure 12 shows the critical 3D and 2D relationships between the bead width (*W*), magnetic field (*M*), and wire feed rate (*F*). It is observed from Figure 11 that the bead width (*W*) increased for all values of *M* from 90 to 150 G for *F* as 6.5 m/min or above. This may have been due to the dominating effect of *HI_f_* for higher wire feed rate (*F*), along with the magnetic field (*M*). The slope of the bead width (*W*) significantly decreased at a 5.5 m/min wire feed rate (*F*) for all values of the magnetic field (*M*). The trend of the plot confirmed the previously discussed behavior of *AR* for the interaction of *M* and *F*. Thus, it was also observed that the direct effect of the wire feed rate (*F*) was the most vital. Still, at the same time, the magnetic field’s (*M*) direct effect and its interaction along with wire feed rate (*F*) could not be ignored.

## 4. Conclusions

- The investigation for the effect of the EMF on the aspect ratio (*AR*) and heat input factor (*HI_f_*) for a magnesium alloy (AZ31B) showed that the effect of the EMF on the arc configuration and arc energy affected the weld quality and the process. The effects of the interaction of the EMF and wire feed rate (*F*) on the heat input factor (*HI_f_*), aspect ratio (*AR*), weld penetration (*P*), and bead width (*W*) were significant. The R-squared value (R^2^) for the aspect ratio (*AR*) was 97.64%, and for the heat input factor (*HI_f_*), it was (94.52%), showing a good correlation for the process.
- The predicted results were in consonance with experimental results using conformity tests. The optimized values of the process parameters for this multi-objective process were 119 G for the magnetic field, 700 mm/min for the welding speed, 5.8 m/min for the feed rate, and a gas flow rate of 11.5 L/min. This resulted in a full weld penetration (*P*) with a corresponding heat input factor (*HI_f_*) of 0.8 and aspect ratio (*AR*) of 2.26.
- The aspect ratio (*AR*), which is a dimensionless number, can be directly associated with the weld quality. A higher value signifies a poor weld penetration (*P*) or a large bead width (*W*). The rise in the magnetic field from 90 to 120 G produced a decline in *AR*. However, a further increase in the magnetic field (*M*) till 150 G caused a sharp increase in the aspect ratio. It was observed that as *M* increased from 90 to 115 G, *AR* decreased for all values of the wire feed rate (*F*). The slope of the aspect ratio (*AR*) was negative for the magnetic field (*M*) until 115 G. However, beyond 115 G, the aspect ratio (*AR*) started increasing and the slope was steeper.
- Like the aspect ratio number (*AR*), the heat input factor (*HI_f_*) may also reflect some information about the weld quality and optimization of the process parameters. Thus, the heat input factor (*HI_f_*), a dimensionless number, similarly shows the cumulative effect of the magnetic field (*M*), wire feed rate (*F*), welding speed (*S*), and gas flow rate (*G*) on the aspect ratio (*AR*) regarding the bead width (*W*) and penetration (*P*). The heat input factor varied from 0.7 to 1.3 for the given range of magnetic fields from 90 to 150 G with a simultaneous variation in the feed rate from 5.0 to 7.0 m/min. For a higher magnetic field (150 G), a frequent arc extinction with a large size spatter and the explosion of melting drops was observed.
- The EMF up to 120 G showed an increase in penetration (*P*) up to the full depth, i.e., 3.7 mm, and supported the arc stability. In comparison, above 120 G, the penetration (*P*) decreased as low as 33% of the sheet thickness (1.0 mm), and frequent arc extinguishing was observed.
- The appearance of weld metals under the EMF was more regular, smooth, and straight than without the EMF.

## Figures and Tables

**Table 1:** Welding parameters and their levels and ranges.

Parameters	Units	−2	−1	0	1	2
*M*	G	90	105	120	135	150
*F*	m/min	5	5.5	6	6.5	7
*S*	mm/min	500	550	600	650	700
*G*	L/min	9	10	11	12	13

**Table 2:** The design and response parameters.

Input Parameters					Responses			
Run Order	*M*	*F*	*S*	*G*	*HI_f_*	*AR*	*P*	*W*
1	105	6.5	650	12	1.14	3.19	2.38	7.6
2	120	6.0	600	11	0.91	3.13	2.36	7.4
3	105	5.5	550	10	0.95	4.50	1.68	7.6
4	120	6.0	600	9	0.75	2.93	2.76	8.1
5	120	6.0	600	11	0.91	3.10	2.58	8.0
6	120	6.0	600	11	0.91	3.45	2.20	7.6
7	120	6.0	600	11	0.91	3.24	2.28	7.4
8	150	6.0	600	11	0.73	4.93	1.48	7.3
9	120	6	700	11	0.785	2.05	3.7	7.6
10	135	6.5	650	12	0.89	3.51	2.22	7.8
11	105	5.5	550	12	0.92	5.83	1.32	7.7
12	120	6.0	600	11	0.85	3.10	2.58	8.0
13	105	6.5	650	10	0.95	2.83	2.82	8.0
14	135	6.5	650	10	0.74	3.09	2.72	8.4
15	120	6.0	600	11	0.92	3.12	2.40	7.5
16	105	6.5	550	10	1.125	2.93	3.00	8.8
17	105	5.5	650	10	0.80	2.74	2.84	7.8
18	120	6.0	600	11	0.92	3.31	2.38	7.9
19	90	6.0	600	11	1.2	3.90	2.00	7.9
20	135	5.5	650	10	0.63	3.50	2.12	7.4
21	135	5.5	550	10	0.74	5.41	1.20	6.5
22	105	5.5	650	12	0.97	3.80	1.92	7.3
23	135	6.5	550	12	1.05	3.78	2.38	9.0
24	105	6.5	550	12	1.35	3.63	2.42	8.8
25	120	6.0	600	13	1.08	4.73	1.52	7.2
26	120	5.0	600	11	0.76	6.50	1.00	6.5
27	120	7.0	600	11	1.07	3.81	2.36	9.0
28	135	5.5	550	12	0.89	6.80	1.00	6.8
29	135	5.5	650	12	0.75	4.78	1.42	6.78
30	120	6.0	500	11	1.1	4.50	1.82	8.2
31	135	6.5	550	10	0.875	3.06	2.94	9.0

**Table 3:** Model validation and confirmation.

Trial Nos.	Process Parameters				Responses							
	*M*	*S*	*F*	*G*	*AR_Pred_*	*AR_obs_*	*HI_f__,Pred_*	*HI* *_f,obs_*	*P_Pred_*	*P_obs_*	*W_Pred_*	*W_obs_*
1.	120	700	6	9	1.74 ± 0.35	2.05	0.63 ± 0.08	0.64	3.4 ± 0.37	3.7	8.0 ± 0.42	7.6
2.	130	600	7	10	3.70 ± 0.35	3.6	0.87 ± 0.08	0.89	1.2 ± 0.37	1.5	5.1 ± 0.42	5.4
3.	150	600	7	10	4.52 ± 0.35	4.2	0.70 ± 0.08	0.77	0.7 ± 0.37	1.2	4.9 ± 0.42	5.0
